# Chinese Character Features Facilitate Working Memory Updating: Evidence From the EEG

**DOI:** 10.1002/brb3.70682

**Published:** 2025-07-10

**Authors:** Hongli Li, Decai Ren, Yihang Ouyang

**Affiliations:** ^1^ School of Education Anyang Normal University Anyang China

**Keywords:** Chinese character, ERP, time‐frequency analysis, working memory updating

## Abstract

**Introduction:**

According to the multicomponent model of working memory (WM), the phonological loop serves to protect WM representations from interference through its phonological storage and rehearsal mechanisms, thereby enhancing performance on WM tasks. However, the neural mechanisms by which language knowledge facilitates WM remain unclear. The present study aims to explore how Chinese character features influence WM and to uncover the underlying mechanisms involved.

**Methods:**

Using the 2‐back task paradigm with both real and pseudo characters, we recorded behavioral and electroencephalogram (EEG) data from 33 participants (aged 18–24 years; 16 males). By employing a combination of event‐related potential (ERP) and time—frequency analysis (TFA) techniques, we investigated differences in neural activity among participants as they engaged in the real character and pseudo‐character 2‐back tasks.

**Results:**

Behavioral results showed that the *d*‐prime was higher and the reaction time was shorter for the real character 2‐back task. The ERP results revealed that the P200 and P300 amplitudes were significantly smaller during the real character 2‐back task than during the pseudo‐character 2‐back task. TFA results revealed that the event‐related desynchronization (ERD) of alpha oscillations was significantly smaller during the real character task than during the pseudo‐character task. There were no differences in theta activity.

**Conclusions:**

The characteristics of Chinese characters influence the WM updating process. The presence of language knowledge in Chinese characters may enhance the brain's ability to perform verbal WM tasks more efficiently. In contrast, the absence of language information in pseudo‐characters adversely affects the continuous updating process of verbal WM.

## Graphical abstract

The findings suggest that the updating of Chinese characters elicits smaller P200 and P300 amplitudes in the parietal region and reduced alpha‐ERD activity in neural oscillations compared to updating pseudo‐characters. These results confirm that the inherent properties of Chinese characters may enhance cognitive efficiency in working memory updating tasks.

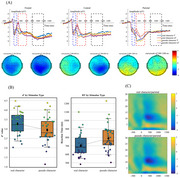

## 1 Introduction

Working memory (WM) is a core cognitive mechanism that plays a crucial role in our cognitive processes. It enables the temporary storage of information, ensuring that key information remains readily available for immediate use. At the same time, it allows us to perform mental operations on this stored information, facilitating tasks such as problem‐solving, decision‐making, and learning (Baddeley 2003, 2012). The WM system is not only used for the temporary storage of information but also allows us to manipulate information in our minds to adapt to new information inputs. This updating mechanism is also a core component of WM and is involved in the completion of multiple cognitive tasks (Linares et al. 2016; Nir‐Cohen et al. 2020).

In the multicomponent WM model (Baddeley and Hitch 1974; Baddeley 2000), WM is conceptualized as a system consisting of two specialized, temporary memory buffer systems (the phonological loop and the visuospatial sketchpad) and a supervisory system (the central executive). The phonological loop is a cyclic rehearsal system for maintaining internal verbal information, and the visuospatial sketchpad is responsible for updating and preserving visual information, and these processes are all regulated by the central executive system (Baddeley 2003). In Baddeley's (2000) WM model, information is represented in different storage subsystems according to the form of the information, such as verbal or visuospatial. The task‐relevant feature in verbal tasks is the identity of the words, while in spatial tasks, the task‐relevant feature is the location (Chen et al. 2008). The completion of verbal WM tasks relies heavily on phonological encoding, while the completion of visuospatial WM usually involves objects that cannot be verbally rehearsed and depends more on the encoding of visual features/spatial locations. In the study of verbal WM and spatial WM, it has been found that the reaction time (RT) in verbal WM tasks is often shorter than that in spatial WM tasks, and in neural activity, more resources for updating may be allocated in the verbal WM task than in the spatial WM task (Shen et al. 2018). This might be because in a relatively well‐developed language processing system, efficient phonological encoding and storage mechanisms can quickly transform the received verbal information into a form that can be processed in WM. However, in verbal WM tasks, how language knowledge promotes the performance of memory updating remains unclear.

Research has shown that the mental images in WM decay rapidly over time, unless the visual information in memory is continuously updated through articulatory rehearsal or by directing attention (Oberauer et al. 2012). A simple yet powerful explanatory model is the rehearsal mechanism of the phonological loop. In visual memory experiments involving linguistic information, since the stimulus materials are often nameable, this allows the subjects to verbally encode the materials, so as to utilize the ability of the phonological loop to store the serial order (Baddeley 2000). Due to the efficiency of phonological storage in serial memory, adult participants usually choose to name and silently recite the visually presented items, thus transferring the information from visual encoding to auditory encoding (Baddeley 2000). When a large amount of information is stored in WM, phonological rehearsal can, to a certain extent, compensate for the decay of memory traces. However, during the operation of WM, it remains unclear to what extent the continuous updating of stimuli depends on language‐related factors.

Therefore, the main issue we are concerned with is whether the WM updating can benefit from the rehearsal of language knowledge. A study by Brady and Störmer (2022) explored whether real‐world objects, colors, perceptually matched objects with less significance, and completely scrambled objects could benefit from deep processing. The results showed that the capacity of visual WM varies depending on the type of stimuli, and more meaningful stimuli are better remembered. Research in the field of verbal WM has also found that there is an interaction between long‐term memory (LTM) knowledge and verbal WM. For example, there are different psycholinguistic effects in verbal WM tasks (Querella and Majerus 2024). The word frequency effect, word imaginability, semantic relatedness effect, or the sentence superiority effect, and so forth, also exist in the WM process (Baddeley 2000; Hulme et al. 1991). In memory sequences, research has found that vocabulary size, word frequency, imaginability, and the ratio of words to nonwords all affect the stability of phonological traces. These factors influence the phonological recognition performance of both words themselves and nonwords (Jefferies et al. 2006a). Savill et al. (2018) investigated the contribution of semantic knowledge to the stability of phonological traces by adding nonwords to mixed lists, and compared the immediate serial recall performance under pure word lists (coherent sequences) and mixed lists containing both words and nonwords (random sequences). The results revealed significant differences in phonological stability between coherent sequences and random sequences. The semantic binding theory also proposes that lexical/semantic factors contribute to binding the phonemes of individual items together, and lexical/semantic factors facilitate phonological coherence during the processes of information encoding, maintenance, and recall (Jefferies et al. 2006b). These studies all demonstrate that language‐related knowledge plays a certain role in WM performance.

In the Chinese language system, Chinese characters have the nature of symbols, which is different from alphabetic writing systems. Unlike alphabetic writing systems that use letter sequences to represent the pronunciation of words, Chinese characters convey meanings through their visual forms, and they usually have complex structures and semantic components (Perfetti and Zhang 1991; Shu et al. 2003). For example, the Chinese character 山 (/shān/, “mountain”) has a shape that resembles several mountain peaks stretching continuously. Just by looking at this character, one can understand that it represents mountains. However, this does not mean that phonetic sounds are insignificant in the processing of Chinese characters. On the contrary, phonetic knowledge plays a crucial role in all aspects of Chinese character processing (Peng and Hui 1997; Perfetti and Zhang 1991; Xu et al. 1999). Therefore, exploring whether there is a language advantage in WM within the framework of the Chinese language system can supplement the conclusions drawn from research on alphabetic WM. In this study, we introduced stimuli of Chinese characters and pseudo‐characters, and compared the differences in neural activities during the WM updating process between Chinese characters and pseudo‐characters.

Pseudo‐characters have structures similar to those of Chinese characters in terms of visual features, but they do not have recognizable phonemes and lack corresponding phonetic information. For example, a pseudo‐character (e.g.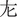) is particularly similar in structural features to Chinese characters (e.g.,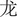, /lónɡ/ or /loong/, “dragon”). Although pseudo‐characters conform to orthographic rules, they cannot be pronounced and do not convey any meaning (Li and Zhao 2024b). This indicates that Chinese characters and pseudo‐character materials have similar structural features visually, yet they do not possess the corresponding linguistic knowledge. In order to distinguish it from the concept of pseudo‐characters, the Chinese characters will be referred to as “real characters” hereinafter.

In the study by Li and Zhao (2024b), pseudo‐character stimuli were used as distractors to explore the impacts of pseudo‐character interference on the WM process and to reveal the influence mechanism of pseudo‐character distractors that are visually similar to Chinese characters on the WM process. After the completion of this study, we considered a question. Since Chinese characters and pseudo‐characters have similar visual representations (but pseudo‐characters lack phonetic and semantic representations), directly comparing the differences between the real characters and pseudo‐characters in the WM task may be more helpful in revealing the influence mechanism of language knowledge (especially phonetic representation) on the WM process. Therefore, in this study, Chinese characters and pseudo‐characters were mainly used as target stimulus materials to compare the differences in neural activity between these two materials during the 2‐back task, aiming to reveal the influence mechanism of language knowledge on the WM process.

Research has shown that when word combinations generate meaning, the phonetic traces that support language are strengthened through the semantic combination process (Jefferies et al. [Link], 2006a; Kowialiewski and Majerus 2020). The contribution of semantics operates at the whole‐word level. For example, the activated semantic and lexical representations enable people to complete the missing parts in the phonetic traces (Savill et al. 2018). During the process of speech production/understanding, the semantic and phonetic patterns corresponding to specific words are correlated, and lexical and semantic knowledge play an important role in the coherence of phonetic representations (Jefferies et al. [Link], 2006a). These research findings indicate that the availability of the phonetic representations of real characters stored in LTM can help characters make better use of the rehearsal mechanism of the phonological loop during the refreshing process. By continuously updating real characters through the phonological loop rehearsal system, it promotes the continuous refreshing operations of real characters in WM. However, due to the lack of corresponding language knowledge, pseudo‐characters find it difficult to rely on the phonetic rehearsal system, which may lead to a lack of phonetic coherence of pseudo‐characters during WM updating and thus result in poor performance. This differential characteristic provides good support for us to explore the influence of language‐related factors of real characters on the WM process. By exploring the possible advantageous effect of phonetic rehearsal in real characters on WM, it reveals the importance of language factors for WM operations.

In the research on the neural mechanisms of WM updating, the *N*‐back task is one of the most widely used experimental paradigms. This task typically involves processes of information manipulation and the storage of information (Jonides et al. 1997). In this task paradigm, a series of items (such as numbers, letters, or locations) is usually presented. The participants are required to determine, when each item is presented, whether the attribute of the current item matches that of the item presented *N* positions earlier (e.g., Jonides et al. 1997; Linares et al. 2016; Ren et al. 2023). In this task paradigm, compared to the internal representation of the stimulus in memory, an event is classified as a match (target trial) or a mismatch (nontarget trial) (Pelegrina et al. 2020). For example, in the 2‐back task, participants are required to delete the previous item, add a new item, and maintain the presentation order of each item as a new item (Shen et al. 2018). Correspondingly, WM updating is not a one‐step replacement but requires at least two steps of shift and replacement operations: After matching with the newly presented item, transfer the current 1‐back item to the 2‐back position, and the current item replaces the content in the 1‐back position (Chen et al. 2008). From the perspective of the time processing sequence, the 2‐back task usually involves different processing stages such as item updating operations and maintenance (Ren et al. 2023). Therefore, it is necessary to pay attention to the neural activity at different stages in the study of the WM.

Previous studies have shown that both the spectral power of the electroencephalogram (EEG) (in particular the theta and alpha bands) and event‐related potentials (ERPs) can be used to measure brain activity in the WM task (Brouwer et al. 2012; Dong et al. 2015). The EEG records the spontaneous rhythmic electrical activity of neurons. In addition, the high temporal resolution of ERP technology makes it possible to separate neural activity at different stages in *N*‐back tasks. This further allows us to study the different cognitive processes that occurred in the WM task (e.g., Chen et al. 2008; Pelegrina et al. 2020; Ren et al. 2023; Shen et al. 2018). In the research exploring the electrophysiological activity during the *N*‐back task, the P200 and P300 are commonly used electrophysiological indices (e.g., Dong et al. 2015; Gómez et al. 2018; Polich 2007; Wongupparaj et al. 2018). The latency of the P200 component occurs at approximately 200 ms and is generally thought to reflect the processes of perceptual matching and stimulus classification, such as stimulus detection, evaluation, storage, and encoding (Wongupparaj et al. 2018). The research on Chinese character recognition has demonstrated that the P200 component may be related to the orthography and phonetic features of extracting words in the early stage of lexical processing (C. Y. Lee et al. 2007). The P300 is a positive ERP component with a relatively long latency, with its peak typically occurring approximately 300–700 ms (Gómez et al. 2018; Polich 2007; Sambo and Forster 2011). The amplitude of the P300 reflects the allocation of attentional resources, the intensity of processing, and the updating of the context (Polich 2007; Vilà‐Balló et al. 2018).

In the study of the *N*‐back task, the slow wave (SW) is an ERP component observed in many studies (Bailey et al. 2016; Li et al. 2024; Ren et al. 2023). The maintenance of information in WM is mainly related to the SW component, which persists during the maintenance interval of information. Peaks of SW occurred approximately 500–1500 ms after the stimulus onset. This component significantly predicts the number of items an individual can maintain in memory (Li et al. 2024; Luria et al. 2016). Studies have shown that SW activity is observed during the response–stimulus interval of the *N*‐back task, which may be related to the maintenance of information between different trials in the task. They found that the SW in both the anterior and posterior regions of the scalp is sensitive to the memory load in the *N*‐back task (Bailey et al. 2016).

The neural oscillation rhythms of the brain also play a central role in integrating brain activities across different regions (Brookes et al. 2011). The event‐related spectral perturbations (ERSP) are the EEG spectral changes of ERPs, which are typically displayed in a two‐dimensional time‐frequency domain. Event‐related synchronization (ERS) and desynchronization (ERD) can be identified from ERSP (Delorme and Makeig 2004). Research has shown that theta (4–8 Hz) and alpha (8–13 Hz) oscillations are crucial for sensory processing and memory (e.g., Gomarus et al. 2006; Zhou et al. 2023). The theta band has been identified as a key component of WM‐related activities (Bastiaansen and Hagoort 2003; Brookes et al. 2011; Gevins and Smith 2000; Gomarus et al. 2006; Sauseng et al. 2010). Some electrophysiological studies have reported that the processing of WM depends on the energy in the frontal midline cortex theta band (e.g., Hsieh et al. 2011; Hsieh and Ranganath 2014; Jensen and Tesche 2002; Ratcliffe et al. 2022; Scheeringa et al. 2009). In addition, alpha oscillations are interpreted as an active mechanism of the inhibitory function of neuronal processes (Gutteling et al. 2022), which is related to the suppression of distracting inputs during WM tasks (Zhou et al. 2023). The regulation of alpha power reflects the active allocation of cognitive resources in the brain. That is, the energy in the alpha frequency band participates in resource allocation by actively inhibiting regions unrelated to the task. Alpha activity encompasses two types of neural oscillation patterns: alpha‐ERS and alpha‐ERD. Alpha‐ERS reflects top‐down, inhibitory control processes that actively suppress neural activity to modulate cognitive processing (Klimesch et al. 2007). This mechanism enables the inhibition of brain regions involved in the processing of irrelevant information to be inhibited, allowing more focused attention to be given to relevant stimuli. In contrast, alpha‐ERD reflects the gradual release of inhibition. During the execution of complex tasks, multiple brain regions become activated to form a complex neural network for processing information. The presence of alpha‐ERD indicates that brain regions that were previously under inhibitory control are being released from inhibition. Their neuronal activity then shifts from a relatively synchronized resting state to a state of task‐specific activity, enabling these regions to participate in complex information processing (Klimesch et al. 2007).

Therefore, this study primarily uses ERP technology to investigate differences in neural activity when processing real and pseudo‐characters. As mentioned before, phonetic coherence is crucial for the updating process of verbal WM. Real characters contain rich knowledge of phonetics and semantics, which can help make better use of the articulatory rehearsal mechanism of the phonological loop and promote performance in the WM task. We hypothesize that real characters with more comprehensive language‐related factors will perform better when updating WM than pseudo‐characters that lack language‐related information. Specifically, compared to pseudo‐characters, real characters may respond faster in WM tasks. At the same time, in time‐domain ERPs, due to the facilitation effect of phonetic knowledge, the amplitudes of P200 and P300 in real character tasks will be smaller. Additionally, given that pseudo‐characters are more difficult to recall than real characters, the pseudo‐character 2‐back task may result in higher theta power and alpha power.

## 2 Methods

### 2.1 Participants

A total of 35 native Chinese speakers were selected as participants for the experiment. Among them, two participants were excluded due to the large amount of artifacts in their EEG data. The data of the remaining 33 participants (16 males) were included in the statistical analysis. Their ages ranged from 18 to 24 years, with a mean age of 20.64 years (SD_age_ = 2.10 years). Sample size calculation was performed using G*Power 3.1 software (Faul et al. 2009). Based on a medium effect size (*f* = 0.25), a significance level of *α* = 0.05, and a statistical power of 0.80, the analysis indicated that a minimum of 27 participants was required for the experiment. The sample size utilized in the current study met these predetermined statistical criteria, ensuring sufficient power to detect meaningful effects and enhancing the reliability of the findings. All participants provided written informed consent prior to their involvement in the study. Following the completion of the experiment, they received appropriate compensation for their participation.

### 2.2 Stimuli and Procedure

#### 2.2.1 Materials

The real character materials were mainly selected from the Chinese character frequency corpus of the Modern Chinese Corpus (Li and Zhao 2024b). For this study, eight real characters with occurrences ranging from 1000 to 10,000 were chosen. The average number of strokes of these real characters was 6.63, with an average number of occurrences was 3819 times and an average frequency rate of 2.47 × 10⁻^2^. Additionally, eight pseudo‐characters were selected as materials for the pseudo‐character 2‐back task. These pseudo‐characters lack both phonetic and semantic information compared to Chinese characters. For example, a pseudo‐character (e.g.,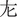) displays structural features that are particularly similar to those of actual Chinese character (e.g.,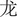, /lóng/ or /loong/, “dragon”), yet they do not possess corresponding phonetic or semantic representations. The average number of strokes among the eight pseudo‐characters was found to be 6.25. Furthermore, visual complexity is indicated by the number of strokes in a character. Thus, we employed an independent samples *t*‐test to conduct statistical analysis on the stroke counts between the two types of materials. The results revealed no significant difference in the number of strokes between the two types of materials, *t*(14) = 0.767, *p* = 0.456.

#### 2.2.2 2‐Back Task

**FIGURE 1:**
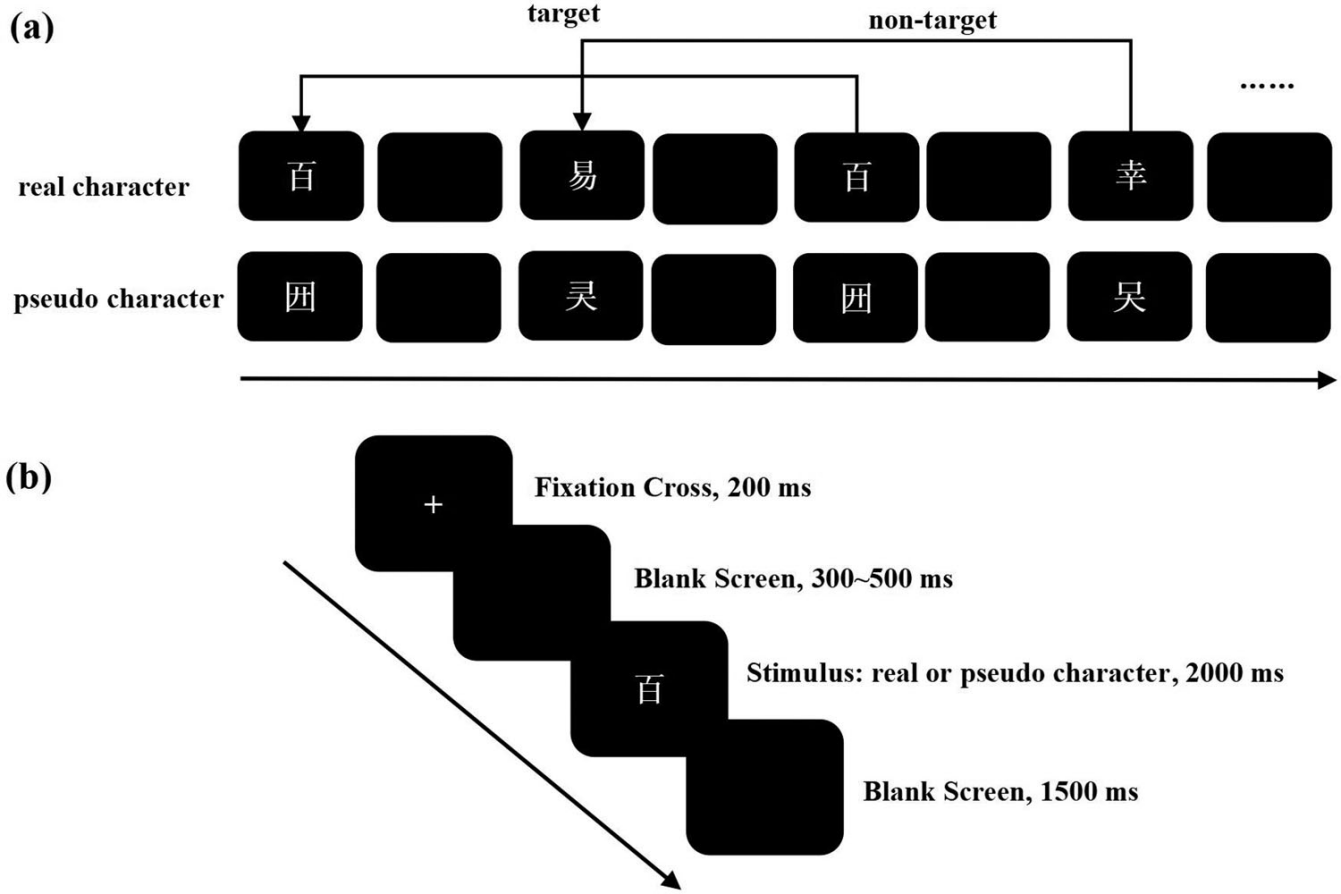
(a) The presentation of materials in the 2‐back task. (Note: The experiment includes the real character 2‐back task and the pseudo‐character 2‐back task.) (b) The experimental procedure of the 2‐back task paradigm.

The 2‐back task paradigm using real and pseudo‐characters (as shown in Figure 1) was mainly adopted to investigate the performance of real characters and pseudo‐characters in the updating process. All experimental procedures were programmed using *E*‐prime 3.0. The participants were seated at a distance of 60–70 cm from the screen, and the eccentricity of the real characters or pseudo‐characters on the horizontal plane was 4.78°. During the experiment, a fixation point “+” would appear at the center of the screen for 200 ms. Then, a randomly jittered blank screen would be presented for 300–500 ms. Immediately after that, the stimulus materials (real characters or pseudo‐characters) would be shown within a 2000 ms response window. Finally, after an interstimulus interval (ISI) of 1500 ms, the next trial would begin. The stimulus materials were composed of eight real characters and eight pseudo‐characters, and each real character or pseudo‐character was presented 30 times during the entire experiment. In the real character 2‐back task, the participants were required to judge whether the currently presented real character was the same as the real character that appeared two steps earlier. If they were the same, the participants should press the “F” key (target stimulus), and if they were different, they should press the “J” key (nontarget stimulus). Similarly, in the pseudo‐character 2‐back task, it is necessary to determine whether the current pseudo‐character was the same as the pseudo‐character that appeared two steps earlier. If they were the same, press the “F” key; otherwise, press the “J” key. Each task condition consisted of four blocks, and each block included 60 trials, among which there were 20 target trials (33.33%) and 40 nontarget trials (66.67%) (Li and Zhao 2024a, 2024b). The entire experimental process had a total of eight blocks, amounting to 480 trials in total. To avoid the influence of fatigue effects and practice effects on the experimental results, we adopted the ABBA counterbalancing design. That is, participants are required to first complete two blocks of the real character 2‐back task, then complete two blocks of the pseudo‐character 2‐back task. For the subsequent four blocks, the order is reversed.

Before the experiment, the participants were required to complete practice tasks. They could only enter the formal experiment if their practice accuracy rate reached more than 70%. The real characters (pseudo‐characters) that appeared in the practice tasks would not be used again in the formal experiment. In addition, the experimental order of the real character and pseudo‐character 2‐back tasks was also balanced among the participants. That is, half of the participants first performed the real character 2‐back task and then the pseudo‐character 2‐back task, while the order for the other half of the participants was exactly the opposite.

### 2.3 EEG Recording

EEG data were acquired continuously with a 64‐channel amplifier (10–20 system) in the eego mylab system (ANT Neuro, the Netherlands). The Cpz electrode was used as an online reference electrode, and the GND electrode was used as a ground electrode. The scalp resistance of all electrode points was maintained below 10 kΩ, and the sampling frequency was set at 500 Hz. The data were analyzed offline using Matlab (Version 2016b) and the EEGLAB toolbox (v14.1.2) (Delorme and Makeig 2004). The data were filtered with a band‐pass filter of 0.1−30 Hz, and the mean of the bilateral mastoids (M1∖M2) was used as an offline re‐reference. The data were segmented from 1000 ms before the stimulus onset to 2000 ms after the stimulus onset. In the ERP analysis, the segments from −200 ms to 1500 ms were extracted (−0.2 to 1.5 s), and the 200 ms period before the stimulus onset was used as the baseline for baseline correction of the segmented data, and the EEG segments with amplitudes exceeding ±100 µV were removed. Independent component analysis (ICA) was employed to eliminate the influence of blink and electro‐oculogram artifacts. Subsequently, the EEG segments of participants under each condition were superimposed, and finally, the average amplitudes under target and nontarget stimuli were calculated to obtain the ERP waveform purely induced by the stimulus conditions.

### 2.4 Statistical Analysis

#### 2.4.1 Behavioral Data

In the experimental tasks, the participants’ discrimination index *d*‐prime (*d′*) and mean RT in each task (real character and pseudo‐character) were used as the dependent variable indicators of the behavioral results of this task. The *d′* was used as indicators to evaluate the sensitivity of participants in distinguishing between targets and nontargets, and at the same time calculate the mean RT of participants in each task. The formula for calculating *d′* is as follows: *d′* = *Z*_Hit_ − *Z*_False Alarms_, where Hit represents the hit proportion (hits/[hits + misses]) when the signal is present, also known as the hit rate, and False Alarms represents the false alarm proportion (false alarms/[false alarms + correct negative]) when the signal is absent, that is, the false‐alarm rate (Haatveit et al. 2010). During the analysis of RT, the trials with incorrect responses, those with a RT less than 150 ms, and those exceeding 3.5 standard deviations of the RT needed to be removed (Li and Zhao 2024b). The paired samples *t*‐test method was adopted to compare the differences in *d′* and RT of the participants under different task conditions.

#### 2.4.2 ERP Data Analysis

Only the trials with correct responses in both target and nontarget trials were included in the analysis, and the number of trials excluded accounted for 0.2% of the total number of trials. Meanwhile, the trials with large artifacts were removed, and the remaining number of valid trials accounted for 77.05% of the total number of trials. After that, the valid trials in the real character 2‐back task and the pseudo‐character 2‐back task were averaged. The average number of valid trials under each experimental condition is shown in Table 1.

**TABLE 1:** The number of valid trials included in each experimental condition (*n* = 33).

	Target	Nontarget
	*M* ± SD	*M* ± SD
Real character	61 ± 13	127 ± 28
Pseudo‐character	57 ± 13	125 ± 23

In order to dynamically assess the neural activity during the character recognition, updating, and maintenance stages, the P200, P300, and SW components were selected as the dependent variables of ERPs. According to previous studies, the earliest positive component was P200, which typically reached its peak approximately in 150–275 ms after stimulation onset (Ren et al. 2023). The peak of the P300 component during the updating stage usually occurred within the time window of 300–600 ms (Li and Zhao 2024a, 2024b). The peak of SW usually occurred within 500–1500 ms after the stimulus onset. Especially during the early maintenance stage, a time window of 800–1200 ms is commonly employed to evaluate the neural activity at this stage (Ruchkin et al. 1995). Based on prior research and visual inspection of the expected time window, we established the analysis time windows as follows: P200 (150–250 ms), P300 (300–600 ms), and SW (800–1200 ms). Regarding the selection of electrode regions, the electrodes in the frontoparietal region were primarily utilized. In the frontal region, the average amplitudes of the three electrode sites (F3, Fz, and F4) were selected. In the central region, the average amplitudes of the three electrode sites (C3, Cz, and C4) were selected. In the parietal region, the average amplitudes of the three electrode sites (P3, Pz, and P4) were selected (e.g., Dong et al. 2015; Morrison and Taler 2020; Vilà‐Balló et al. 2018). For each dependent variable, a repeated measures analysis of variance (rm ANOVA) with a 2 (stimulus type: real character and pseudo‐character) × 2 (trial type: target and nontarget) × 3 (electrode region: frontal, central, and parietal) design was used for statistical analysis.

#### 2.4.3 Time‐Frequency Analysis (TFA)

The calculation window for TFA was set from 1000 ms before the stimulus onset to 2000 ms after the stimulus onset (−1000 to 2000 ms). In the TFA, the Hanning taper method of the short‐time Fourier transform was used. The frequency range was set from 1 to 30 Hz, with a step size of 1 Hz. The length of the time window used is 500 ms. The time window will slide from −1000 ms to 2000 ms with a step size of 50 ms. The overlap duration between adjacent windows is 450 ms, and the overlap ratio is 450/500 = 90%. These calculations were all performed using the FieldTrip toolbox (Oostenveld et al. 2011) and run on the Matlab software. The obtained values of oscillatory energy were baseline‐corrected using the period from 750 to 250 ms before the stimulus onset (−750 to −250 ms) as the baseline (Li et al. 2024) and then converted into decibel scales (10 × log [µV^2^]). The TFA windows were mainly based on the time windows of the time‐domain ERP analysis, and mainly focused on analyzing the theta (4–8 Hz) frequency band and the alpha (8–13 Hz) frequency band at different time windows of each electrode region. A 2 (stimulus type: real character and pseudo‐character) × 3 (electrode region: frontal, central, and parietal) rm ANOVA was used to conduct statistical analysis on the above‐extracted dependent variable indicators.

When the results of the statistical analysis showed non‐sphericity, the Greenhouse–Geisser method was employed to correct the *p*‐values of the rm ANOVA. Moreover, the Bonferroni method was used for correction in multiple comparisons.

## 3 Results

### 3.1 Behavioral Results

**FIGURE 2:**
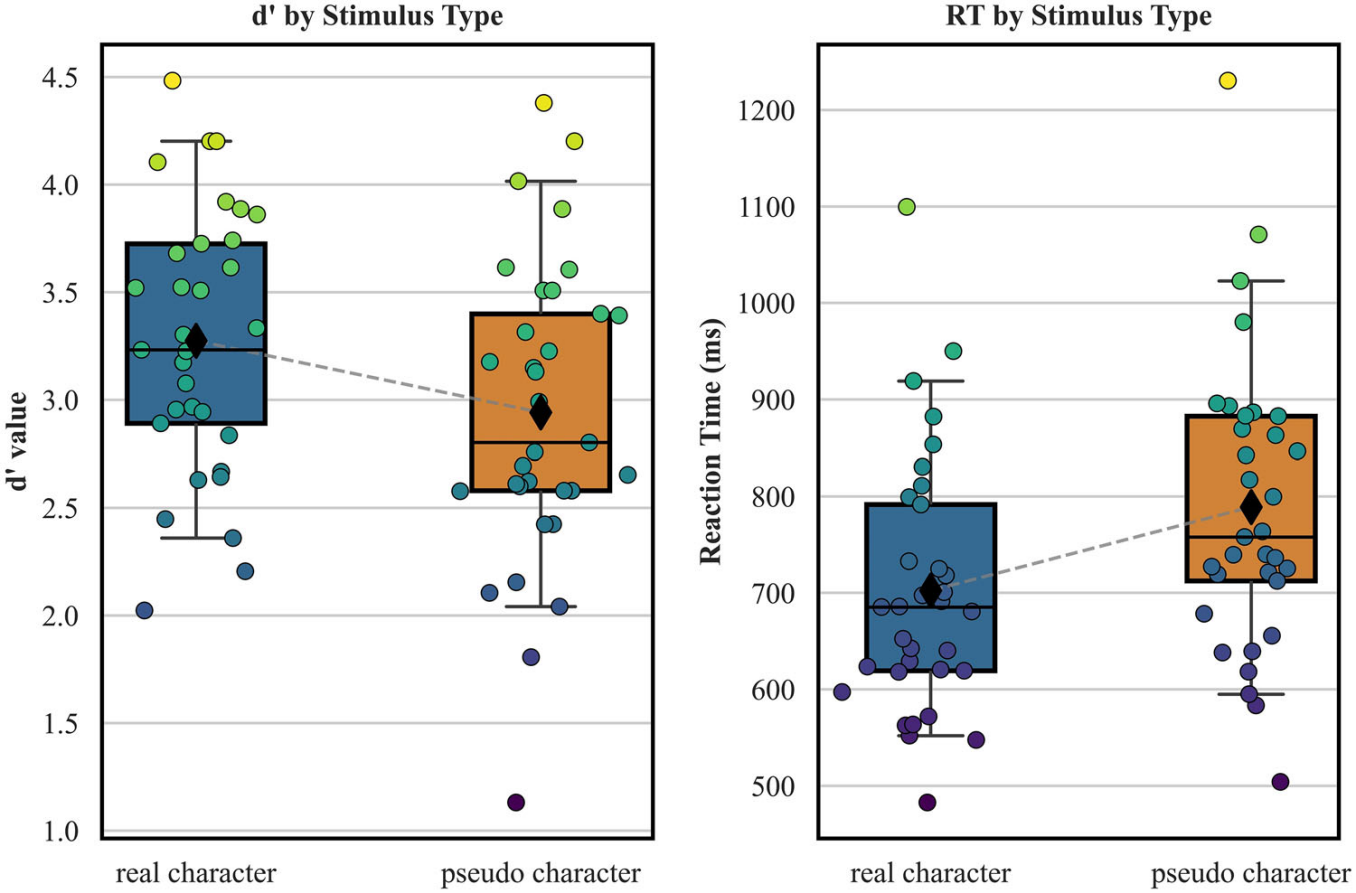
The *d′* and RT in real character and pseudo‐character 2‐back tasks.

As shown in Figure 2, in terms of the discriminability index *d′*, the *d′* value in the real character 2‐back task (*M* = 3.27, SD = 0.61) was higher than that in the pseudo‐character 2‐back task (*M* = 2.94, SD = 0.71), *t*(32) = 4.810, *p* < 0.001, 95% CI = [0.19, 0.47], Cohen's *d* = 0.50. The mean RT in the real character 2‐back task (*M* = 702 ms, SD = 134 ms) was shorter than that in the pseudo‐character 2‐back task (*M* = 789 ms, SD = 151 ms), *t*(32) = −7.317, *p* < 0.001, 95% CI = [−110.86, −62.58], Cohen's *d* = −0.62.

### 3.2 Event‐Related Potentials

**FIGURE 3:**
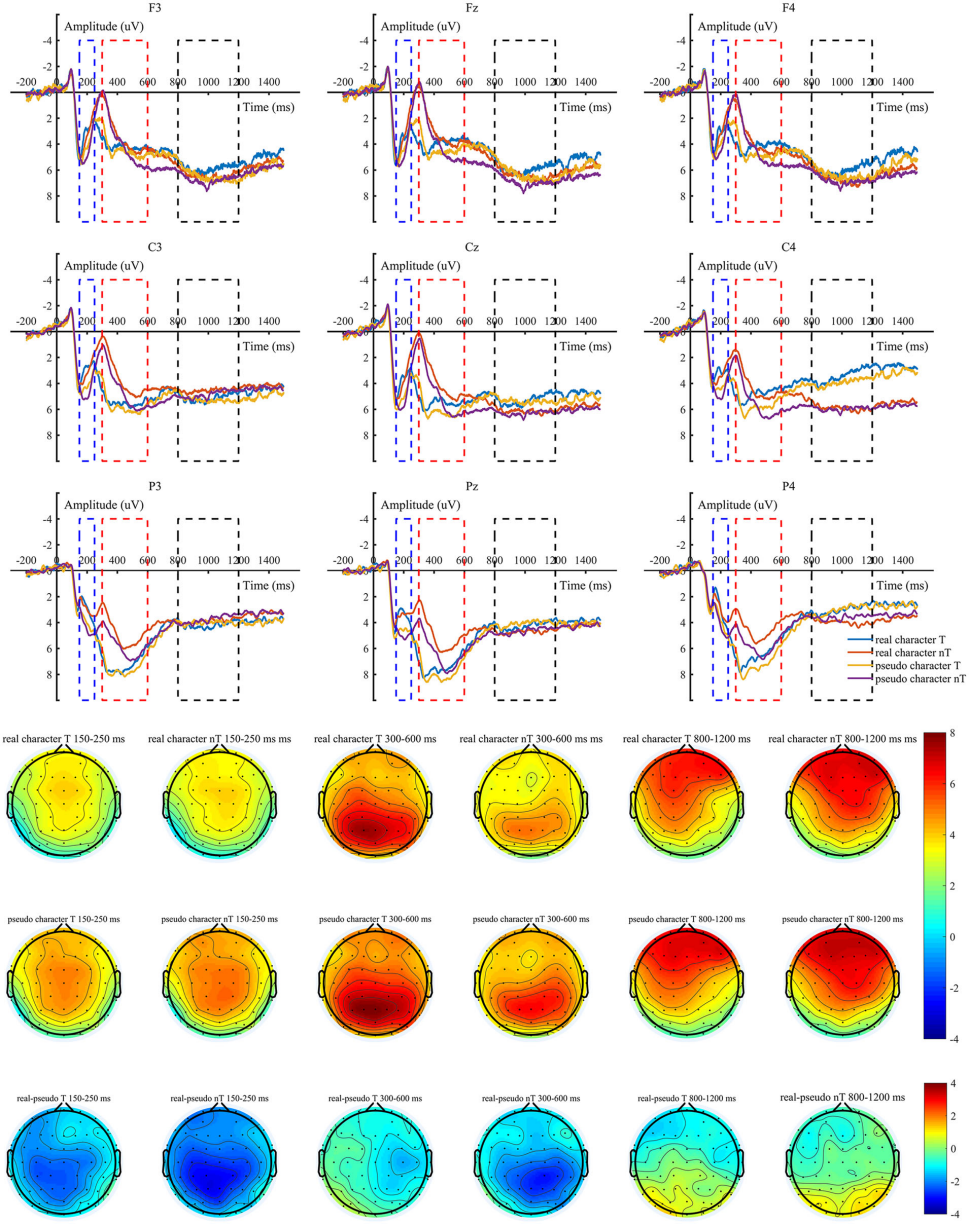
The P200, P300, and SW waveforms at the frontal, central, and parietal regions during real character and pseudo‐character 2‐back tasks (Note: The ERPs induced an P200 [first blue dashed area in the ERP plot], an P300 [second red dashed area in the ERP plot], and an SW [third black dashed area in the ERP plot], T = target.).

The results of the rm ANOVA for the P200, P300, and SW amplitudes in different brain regions during the real character and pseudo‐character tasks are detailed in Table 2. Figure 3 shows the waveforms and brain topographies under various conditions.

**TABLE 2:** rm ANOVA result for stimulus type (real character and pseudo‐character), trial type (target and nontarget), and electrode region (frontal, central, and parietal) for P200, P300, and SW.

	P200 (150–250 ms)				P300 (300–600 ms)			SW (800–1200 ms)		
	*F*		*p*	*η* ^2^_p_	*F*	*p*	*η* ^2^_p_	*F*	*p*	*η* ^2^_p_
a	71.524	0.000		0.691	8.381	0.007	0.208	0.383	0.541	0.012
b	0.950	0.337		0.029	16.959	0.000	0.346	2.055	0.161	0.060
c	0.275	0.760		0.009	11.172	0.000	0.259	4.182	0.020	0.116
a × b	1.010	0.322		0.031	1.857	0.183	0.055	0.084	0.774	0.003
a × c	4.301	0.018		0.118	1.774	0.178	0.053	4.222	0.019	0.117
b × c	1.345	0.268		0.040	21.870	0.000	0.406	1.549	0.220	0.046
a × b × c	0.147	0.864		0.005	3.614	0.033	0.101	0.075	0.928	0.002

*Note*: a = task type (real character task and pseudo‐character task), b = trial type (target and nontarget), c = electrode region (frontal, central, and parietal).

#### 3.2.1 P200

The main effect of stimulus type was significant, *F*(1, 32) = 71.524, *p* < 0.001, *η*_p_^2^ = 0.691. The P200 amplitude in the real character 2‐back task (*M* = 3.27 µV) was smaller than that in the pseudo‐character 2‐back task (*M* = 4.36 µV,* p* < 0.001, 95% CI = [−1.35, −0.83]). The main effect of trial type was not significant, *F*(1, 32) = 0.950, *p* = 0.337. The main effect of electrode region was not significant, *F*(2, 64) = 0.275, *p* = 0.760. The interaction between stimulus type and electrode region also reached a significant level, *F*(2, 64) = 4.301, *p* = 0.018, *η*_p_^2^ = 0.118. The results of simple effect analysis (as shown in Figure 3) indicated that the P200 amplitude of the real character 2‐back task was smaller than that of the pseudo‐character 2‐back task in the frontal, central, and parietal regions (all *p* < 0.001). In short, in the frontal–central–parietal region, the real character 2‐back task activated with a smaller P200 amplitude compared to the pseudo‐character 2‐back task.

#### 3.2.2 P300

For the P300, the results of rm ANOVA showed that the main effect of stimulus type was significant, *F*(1, 32) = 8.381, *p* = 0.007, *η*_p_^2^ = 0.208. The P300 amplitude in the real character 2‐back task (*M* = 4.76 µV) was smaller than that in the pseudo‐character 2‐back task (*M* = 5.49 µV,* p* = 0.007, 95% CI = [−1.24, −0.22]). The main effect of trial type was significant, *F*(1, 33) = 16.959, *p* < 0.001, *η*_p_^2^ = 0.346. The P300 amplitude under the target trial condition (*M* = 5.75 µV) was larger than that under the nontarget trial condition (*M* = 4.50 µV, 95% CI = [0.64, 1.88]). The main effect of electrode region was significant, *F*(2, 64) = 11.172, *p* < 0.001, *η*_p_^2^ = 0.259. The results of post hoc comparisons showed that the P300 amplitude in the parietal region (*M* = 6.40 µV) was larger than that in the frontal region (*M* = 4.00 µV,* p* = 0.004, 95% CI = [0.67, 4.13]) and the central region (*M* = 4.99 µV,* p* = 0.007, 95% CI = [0.33, 2.50]). These results indicated that a strong P300 effect has emerged in the parietal region. The interaction among stimulus type × trial type × electrode region reached a significant level, *F*(2, 64) = 3.614, *p* = 0.033, *η*_p_^2^ = 0.101. The results of simple effect analysis showed that under the target trial condition, there was no significant difference in the P300 amplitude between the real character and the pseudo‐character tasks in all electrode regions (all *p* > 0.05), and under the nontarget trial condition, the P300 amplitude in the real character task was significantly smaller than that in the pseudo‐character task in the central and parietal regions (all *p* < 0.05).

#### 3.2.3 SW

As shown in Figure 3, the main effects of both stimulus type and trial type did not reach a significant level, with *F*s < 2.055 and *p*s > 0.05. The main effect of electrode region was significant, *F*(2, 64) = 4.182, *p* = 0.020, *η*_p_^2^ = 0.116. The interaction between stimulus type and electrode region was also significant, *F*(2, 64) = 4.222, *p* = 0.019, *η*_p_^2^ = 0.117. The results of the simple effects analysis showed that, in the pseudo‐character condition, the SW amplitude in the central region (*M* = 5.18 µV) was significantly larger than in the parietal lobe region (*M* = 3.94 µV, *p* = 0.026, 95% CI = [0.14, 2.70]).

### 3.3 Time‐Frequency Analysis

**FIGURE 4:**
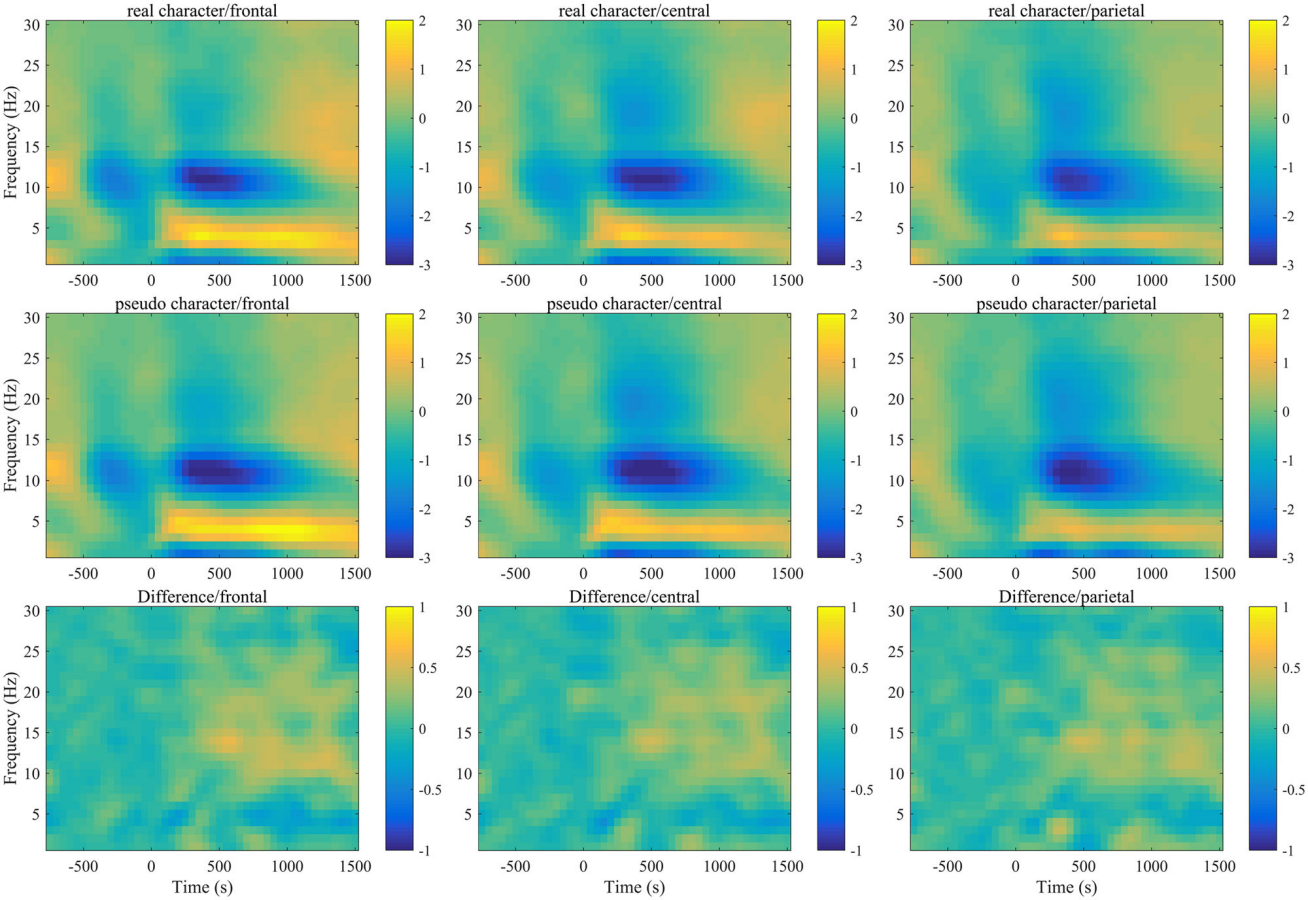
ERSP results for theta band (4–8 Hz) and alpha band (8–13 Hz) in real character and pseudo‐character 2‐back tasks (Note: The first row and second row present the time‐frequency power plots for both the real character and the pseudo‐character 2‐back tasks. The third row presents the time‐frequency power plots for the difference between the real character and the pseudo‐character conditions. The *x*‐axis represents the time window [−750 to 1500 ms], while the *y*‐axis indicates the frequency [1–30 Hz]. The frequency range of 4–8 Hz corresponds to the theta band, and the 8–13 Hz range corresponds to the alpha band. The color bar displays the power ratio in decibel [dB].).

An rm ANOVA was performed on the ERSP results (as shown in Figure 4) in the real character and pseudo‐character 2‐back tasks. The results showed that in the early theta band, the main effect of stimulus type was not significant, *F*(1, 32) = 0.097, *p* = 0.758. The main effect of electrode region was significant, *F*(2, 64) = 19.187, *p* < 0.001, *η*_p_^2^ = 0.375. The post hoc comparison results showed that the theta ERS in the frontal region (*M* = 0.69 dB) and the central region (*M* = 0.65 dB) was higher than that in the parietal region (*M* = 0.18 dB). The interaction between stimulus type and electrode region did not reach a significant level, *F*(2, 64) = 1.091, *p* = 0.342. In the results of the ERSP of the late theta band, it was also found that only the main effect of electrode region reached a significant level, *F*(2, 64) = 17.111, *p* < 0.001, *η*_p_^2^ = 0.348. The post hoc comparison results showed that the theta power in the frontal region (*M* = 0.46 dB) and the central region (*M* = 0.23 dB) was larger than that in the parietal region (*M* = −0.15 dB).

The results of the ERSP of the early alpha band showed that the main effect of stimulus type was not significant, *F*(1, 32) = 2.297, *p* = 0.139. The main effect of electrode region was not significant either, *F*(2, 64) = 0.078, *p* = 0.925. The interaction between stimulus type and electrode region did not reach a significant level, *F*(2, 64) = 0.003, *p* = 0.997. The results of the ERD of the late alpha power showed that the main effect of stimulus type reached a significant level, *F*(1, 32) = 4.953, *p* = 0.033, *η*_p_^2^ = 0.134. The alpha‐ERD in the real character 2‐back task (*M* = −2.25 dB) was smaller than that in the pseudo‐character 2‐back task (*M* = −2.40 dB, *p* = 0.033, 95% CI = [0.01, 0.30]). The main effect of the electrode region was not significant, *F*(2, 64) = 0.443, *p* = 0.644. The interaction between stimulus type and electrode region did not reach a significant level, *F*(2, 64) = 0.914, *p* = 0.406.

## 4 Discussion

In this study, we investigated the electrophysiological manifestations of the influence of phonetic knowledge on WM updating processing by comparing the differences in neural activities during the processing of the 2‐back task for real characters and pseudo‐characters. The results showed that, compared to the pseudo‐character 2‐back task, the *d′* was larger and the RT was shorter in the real character 2‐back task. Electrophysiological activity revealed that smaller P200 and P300 amplitudes were observed during the real character task than during the pseudo‐character task. Additionally, smaller alpha‐ERD activity was generated during the real character WM updating.

Behavioral results indicate that participants were better able to discriminate target stimuli in the real character task than in the pseudo‐character task. Furthermore, RTs were significantly shorter in the real character 2‐back task than in the pseudo‐character 2‐back task. This result confirms that in the Chinese character system, the processing of WM is also affected by the phonological superiority effect. On the one hand, in the 2‐back task, the relatively coherent phonetic encoding in real characters supports the brain to more effectively use the phonological loop to complete the continuous updating operations in WM. However, the corresponding phonetic encoding is missing in the information of pseudo‐characters, making it difficult to rely on the rehearsal mechanism of the phonological loop during the updating process, resulting in lower updating efficiency. In the WM system, the capacity of WM is generally considered to only accommodate a limited number of information chunks, and the duration of information retention is relatively short. If the information is not rehearsed or further processed, the memory trace will decay rapidly over time (Oberauer and Kliegl 2006). According to the energy decay model, due to the limited capacity of memory, memory traces will disappear in a short period of time. In this energy decay model, decay can be overcome through some form of articulatory rehearsal (Hulme et al. 1997). The automatic encoding of real character pronunciation forms an advantage during the WM updating. On the other hand, more meaningful stimuli can benefit from a high‐capacity verbal LTM system or a more accessible LTM (Brady and Störmer 2022). In the 2‐back task, participants are required to continuously compare whether the current stimulus is consistent with the second stimulus presented previously (e.g., Chen et al. 2008; Ren et al. 2023). During the comparison and matching process of this task, real characters, as the stimulus materials, can automatically activate the language knowledge in LTM. In contrast, pseudo‐characters cannot activate the corresponding language knowledge in LTM, resulting in the need to analyze more visual detail features during the matching process. As a result, the updating process of pseudo‐characters has lower discrimination ability and requires a longer RT.

In terms of the time‐domain ERP results, it was found that compared to the pseudo‐character 2‐back task, the real character 2‐back task exhibited smaller amplitudes of the P200 and P300 components. In the field of ERP research on real character recognition, the P200 amplitude is generally considered to be related to the early visual feature encoding and matching processes in real character recognition. The smaller P200 amplitude in the real character task may indicate that the phonetic activation of real characters increases the feedback of sub‐lexical units, thereby making the overall processing of real characters simpler. In contrast, since it is more difficult to activate the phonetic information of pseudo‐characters, the difficulty of processing pseudo‐characters increases. In addition, this processing difference may also affect the activities during the 2‐back task. That is, the facilitating effect of phonetic information on real characters promotes a simpler updating operation of characters in the 2‐back task. However, the lack of phonetic information activation in pseudo‐characters leads to an increase in the difficulty of recognition, which further increases the difficulty of the updating operation in the 2‐back task.

In the WM updating process, especially under the condition of nontarget trials where information needs to be updated, the results showed that the P300 amplitude in the pseudo‐character updating task was larger than that in the real‐character updating task. For proficient Chinese readers, when encountering the operation of continuously updating real characters, they will automatically activate the corresponding phonetic encoding to better complete the updating of real character information through the rehearsal system of the phonological loop. For pseudo‐characters without phonetic and semantic information, participants cannot call on a reliable phonetic rehearsal mechanism. During the information updating process, they need to suppress the visual interference caused by the incomplete structure of pseudo‐characters and require more attentional resources to complete the updating operation of pseudo‐characters, resulting in a larger amplitude of the P300. Therefore, during the real character updating process, participants can more effectively use neural resources to focus their attentional resources on the continuous updating of stimuli. In contrast, during the pseudo‐character updating process, participants may need more attentional resources to recognize, compare, and match pseudo‐character stimuli, resulting in a larger P300 amplitude during the updating process.

Consistent with the findings of previous studies (e.g., Li and Zhao 2024a, 2024b; Pelegrina et al. 2020; Ren et al. 2023), this study also revealed that the P300 amplitude of target stimuli was larger than that of nontarget stimuli. The P300 component is a core indicator reflecting the updating of WM. The amplitude of P300 usually reflects the allocation of attention resources and situational updating (Pelegrina et al. 2020; Polich 2007). In the *N*‐back task, the P300 amplitude decreases as the WM demand increases (Pelegrina et al. 2020). Compared to the repeated stimuli that appear in the target trials, the updating process in which new stimuli replace old stimuli in the nontarget trials requires the involvement of more attention resources. Therefore, it elicits a larger amplitude of the P300. Additionally, this component usually varies with the novelty of the stimulus (Chen et al. 2008). The ratio of stimulus occurrence is typically inversely proportional to the P300 amplitude. That is, the lower the probability of a stimulus appearing, the larger the P300 amplitude (e.g., Pelegrina et al. 2020; Rac‐Lubashevsky and Kessler 2019). In the current experiment, the probability of the stimulus in the target trials was low (1/3 target vs. 2/3 nontarget). The P300 amplitude is primarily attributed to the neural processes underlying the updating of mental representations of the stimulus environment. The main explanation for the P300 amplitude is that it reflects the brain activity when the mental representation of the stimulating environment is updated (Polich and Criado 2006). If a novel stimulus is processed, the attentional mechanism will engage in the cascading update of the memory representation of the stimulus situation, thereby triggering P300. Compared to the repeated stimuli, the emergence of novel stimuli consumes more attentional resources, resulting in a smaller P300 amplitude. Therefore, the appearance of new stimuli in nontarget trials may have elicited a smaller P300 amplitude (Li et al. 2024; Polich and Kok 1995).

In terms of TFA, the results showed that there were differences in alpha power between real and pseudo‐characters during the WM updating, while no significant differences were found in theta power. This may be because in the 2‐back task, the real characters and the pseudo‐characters need to be maintained during the serial updating, and there may be no difference in the demand for WM capacity. Therefore, similar theta power was generated in the WM tasks of the two types of stimuli. In terms of the theta frequency band, the theta power in the frontal and central regions was greater than that in the parietal regions. This result is consistent with previous research conclusions on theta power in the *N*‐back task, that is, the EEG signals within the theta power range are the largest in the frontal midline region of the scalp (frontal midline theta) (Gevins and Smith 2000; Hsieh and Ranganath 2014). When performing the *N*‐back task, theta oscillations may be responsible for regulating the activation of relevant information maintained in WM (H. Lee et al. 2005). The increase in theta oscillations reflects the increase in cognitive effort invested by individuals in the task. More specifically, these efforts may be invested in operations related to serial retrieval and comparison in WM (Gomarus et al. 2006). The greater theta oscillations in the frontal and central regions in this study indicate that the WM updating process may be more dependent on the theta oscillations in the anterior part of the brain. This is because the mental operations of visual stimuli can be carried out in the occipital‐parietal region, but the initiation of these processes requires the central executive/attention control in the frontal region of the brain (Sauseng et al. 2005).

In terms of neural activity in the alpha band, the results showed that the ERD of alpha oscillations during the real character WM updating was smaller than that during the pseudo‐character WM updating. Decreases in alpha power, especially in the higher frequency range, are closely associated with active cognitive processing (Klimesch et al. 2000; Klimesch et al. 2007). In this study, a larger alpha‐ERD was observed in the pseudo‐characters 2‐back task. This is because pseudo‐characters present a greater memory challenge and require more brain resources and effort. This stronger goal‐directed attention causes the brain to allocate more resources to the relevant brain regions, thus eliciting a greater alpha‐ERD response. According to the resource limitation theory, when a task requires more cognitive resources to be accomplished, the brain activates task‐relevant brain regions by adjusting the pattern of neural activity. In the WM task, processing pseudo‐characters demands more cognitive resources than processing Chinese characters. Consequently, a large number of neurons no longer oscillate synchronously when processing pseudo‐characters, resulting in a larger alpha‐ERD. In addition, the study found that the alpha activity reached its maximum desynchronization approximately 400–500 ms after the stimulus, which reflects the actual start of the semantic activation process (Klimesch et al. 2000). The obvious desynchronization of alpha waves is usually related to the activation of semantic LTM. In high‐demand WM tasks, the increase in amplitude is interpreted as the active inhibition of neural circuits serving LTM (Klimesch et al. 1999). The appearance of desynchronization activity indicates that during the information processing, a large number of neurons no longer oscillate synchronously, that is, they are no longer bound by alpha oscillations. The larger alpha‐ERD activity during the updating of pseudo‐characters may indicate that language information in LTM cannot be accessed during the pseudo‐character updating process, which also leads to greater inhibition of neural activities during the pseudo‐character updating operation. Therefore, this also indicates that the ability to effectively activate language‐related information from LTM has a certain impact on the efficiency of neural activities during WM updating.

Overall, the findings related to behavior, ERP (P200 and P300), and alpha‐ERD suggest that the language knowledge of Chinese characters plays a significant role in enhancing both the recognition and updating stages of WM. These results may be attributed to the rehearsal mechanism inherent in the phonological loop. The phonological loop is a relatively modular system that includes a brief storage and a means of maintaining information by vocal or silent rehearsals. Within the framework of WM models, one perspective posits a direct connection between the phonological loop and LTM. Information can flow from the LTM to the phonological loop and vice versa. The phonetic representation associated with Chinese characters facilitates individuals’ more effective utilization of this mechanism. Furthermore, the episodic buffer not only acts as a buffer store, between the components of WM, but also serves as a connector connecting WM and LTM (Baddeley 2012). By combining visual glyphs with phonetic information in the episodic buffer system, it enhances individuals’ ability to swiftly execute recognition and continuous update operations involving real characters during WM tasks. However, due to insufficient language information representation, pseudo‐characters may hinder individuals from leveraging the coding advantages provided by the phonological loop, thereby diminishing their efficiency in completing WM tasks. Consequently, we infer that “the facilitating effect” of language knowledge on task completion in WM can be attributed to both the rehearsal mechanism of the phonological loop and its effective binding capacity for glyph–phonetic information formed within the episodic buffer. This synergy enables individuals to effectively harness language knowledge stored in the LTM while executing verbal WM tasks more efficiently.

Although this study reveals the influence of language knowledge on neural activity in different processes of WM, there are still some shortcomings. First, although in this study, we adopted multiple indicators (P200, P300, SW, theta, and alpha oscillations) to measure the neural activities at different stages in the 2‐back tasks of real characters and pseudo‐characters, there are still certain limitations. Further research can still consider combining different neural indicators, such as N200, to comprehensively elucidate how language knowledge influences neural dynamics across different stages of WM. Additionally, incorporating the examination of beta or gamma band oscillations has the potential to uncover novel and significant findings, thereby enhancing our understanding of the intricate relationship between language knowledge and neural activity during WM tasks. Second, the results of this study confirm that even in structures with similar visual features, compared to pseudo‐characters without language information, language knowledge in characters can help Chinese characters to be better recognized and updated. However, Chinese characters have unique multiple features of shape, phonetics, and semantics. Although the comparison between real and pseudo‐characters can reveal the influence of language knowledge on neural activity during WM processing, it is rather difficult to discriminate meticulously whether the superiority effect of Chinese characters in the WM task is due to phonetic, semantic, or interactive phonetics and semantics. Future research should consider starting from the phonetic features of Chinese characters in a single dimension. In the future, using more rigorous designs, it is necessary to further clarify whether the superiority effect of Chinese characters is reflected in the phonetic or semantic system of Chinese characters, or is the result of the interaction between the phonetic and semantic systems. Third, this study used the 2‐back task paradigm combined with EEG technology, and it could only partially explain the patterns of neural activity involved in updating WM. Measuring WM updating through this task reflects certain characteristics of WM as a whole. However, it does not provide detailed insights into the neural activation patterns corresponding to the different WM subprocesses, such as encoding and maintenance. Further research is necessary to design experimental tasks that encompass these subprocesses and reveal the neural activity of WM subprocesses, as well as to determine at which stage language knowledge influences WM processing.

## 5 Conclusions

In summary, the results of this study indicate that WM updating is also dependent on the phonetic information in Chinese character processing. Skilled phonetic knowledge can help the brain use neural resources more effectively to complete the operation of WM updating. Even in the WM tasks presented visually, Chinese character stimuli can effectively utilize the advantages of phonetic encoding to facilitate the updating operation in WM through continuous rehearsal. Although the visual structure of pseudo‐characters is similar to that of Chinese characters, the lack of phonetic information in pseudo‐characters may affect the continuous updating of information in WM.

## Author Contributions

**Hongli Li**: conceptualization, formal analysis, writing–original draft, writing–review and editing, investigation, methodology, data curation. **Decai Ren**: investigation, data curation. **Yihang Ouyang**: data curation.

## Ethics Statement

All procedures performed regarding human participants were following the ethical standards of the institutional and/or national research committee and with the 1964 Helsinki Declaration and its later amendments or comparable ethical standards.

## Consent

Informed consent was obtained from all participants in the study. Participants consented to publication.

## Conflicts of Interest

The authors declare no conflicts of interest.

## Peer Review

The peer review history for this article is available at https://publons.com/publon/10.1002/brb3.70682

## Data Availability

The data that support the findings of this study are available on request from the corresponding author. The data are not publicly available due to privacy or ethical restrictions. Data were analyzed using EEGLAB (v14.1.2) under the 2016b version of MATLAB and SPSS (IBM, Armonk, NY, USA). This study's design and its analysis have been pre‐registered (Registration DOI: https://doi.org/10.17605/OSF.IO/4DNMK).
